# Editorial

**Published:** 1875-03

**Authors:** 


### Editorial.

***Rush Medical College.***

The Annual Commencement of Rush Medical College was held at Central Hall on the evening of February 10th. The Degree of Doctor of Medicine was conferred by President J. W. Freer upon seventy-seven candidates, whose names are subjoined. The Valedictory Address of the Faculty was delivered by Prof. Henry M. Lyman, and that of the Class by Dr. Henry Fritcher, one of the graduates.

An elegant gold-headed cane was presented, with an appropriate address by Dr. D. Ernest Sedgwick, on behalf of the class, to Prof. Jos. P. Ross, who responded in a very feeling manner.

The Faculty, Alumni and many friends of the College have great cause for congratulation in the large number of students who have been attracted by the determined efforts which they have made during the last four years to elevate and maintain the standard of professional education, and the time-honored reputation of the school, in spite of material obstacles which, to most men, would have seemed insurmountable. The Faculty have endeavored to supply the absence of imposing buildings and many of their comfortable accessories by an increase in the strength and efficiency of their corps of instructors, in the accuracy and comprehension of the system of instruction, and in the exercise of fidelity and zeal in imparting it. They could ask no better evidence that their efforts have been appreciated than that supplied by the presence of so large a number of students, and by the uniform diligence which they have exhibited in the pursuit of their studies.

The class of 1874 and 5, stands in no respect below its predecessors, in intelligence, in preliminary acquirements, and in studiousness. Some, we feel sure, and many, we hope, are destined to fill high positions in the ranks of their profession.

MEMBERS OF THE GRADUATING CLASS.

W. T. ADAMS.
T. L. ASHBAUGH.
S. L. BAUGH.
S. H. BELL.
J. G. BERRY.
A. H. BILL.
J. H. BINNIE.
J. B. BLUE.
N. D. CAMPBELL.
H. A. CLARKE.
R. D. CLARKE.
W. B. CALDWELL.
J. H. CRAIG.
I. H. CADWALLADER.
E. A. CARPENTER.
T. H. CORNWALL.
M. CASSINGHAM.
D. A. DRENNAN.
E. H. DUDLEY.
CHAS. EGAN.
W. C. EGAN.
M. L. FULLENWIDER.
L. M. FOCHT.
G. W. FARROW.
L. A. H. FRIEDERICHS.
H. FRITCHER.
L. M. GIFFIN.
H. L. HARRIS.
T. E. HALL.
J. L. HILL.
R. G. HEALEY.
WM. HUTCHINSON.
R. W. HOYT.
H. L. HARRINGTON.
J. S. KAUFFMAN.
H. B. LOSEY.
E. H. LOCKWOOD.
W. F. LEWIS.
G. D. LADD.
E. M. LANDIS.
O. J. LOWRY.
G. W. McKINNEY.
C. A. McDONELL.
C. MANTOR.
J. D. MANDEVILLE.
D. D. MARR.
T. M. MICHAELS.
W. W. MULLIKEN.
T. C. McCLEERY.
J. J. McFADDEN.
F. H. MORRICAL.
J. A. NOWLAN.
F. J. POPE.
J. PEHRSOON.
J. P. PARKS.
W. G. PUTNEY.
GEORGE RILEY.
A. J. RYAN.
F. REYNER.
W. F. REYNOLDS.
L. C. SEELEY.
D. E. SEDGWICK.
W. W. SQUIRE.
CHAS. SCOTT.
A. T. STEELE.
G. F. SCHREIBER.
F. TURNER.
A. D. TAYLOR.
G. T. THOMAS.
J. W. TRIMMER.
J. H. THOMPSON.
F. J. WILKIE.
W. H. WATSON.
L. R. WILLIAMS.
S. S. WEIDNER.
A. L. R. WHEELER.
G. W. WHEELAND.

HONORARY DEGREE.

Prof. ALBERT SMITH, MD., LL.D., Peterborough, N. H.

*Ad Eundem*—J. C. JOHNSTON.

We cannot better commend to our readers a new and excellent enterprise, in the field of Medical Journalism, than by subjoining, in full, Messrs. G. P. Putnam's Sons' announcement of "A Series of American Clinical Lectures," which speaks for itself.

The first lecture of the series, that of Prof. Sayre, on Diseases of the Hip-Joint, has already appeared and fulfills the promises of the editor and publishers and the expectations of readers. We sincerely hope that the enterprise will meet with such encouragement as will justify its continuance and extension into a wider field.

H.

A SERIES OF AMERICAN CLINICAL LECTURES.

G. P. Putnam's Sons have the pleasure of announcing to the Medical profession that they purpose to commence the publication of a series of *Clinical Lectures* by representative American Medical Teachers, upon topics of practical interest.

The enterprise has been suggested by the Volkmann "Sammlung klinischer Vortrage" (Collection of Clinical Lectures), which has met with great success, and fills an important place in the Medical literature of Germany, and the special value of which is well known to many in this country.

It is intended to select for publication in the series only lectures by recognized Medical instructors; either Professors in Colleges, or Hospital Physicians, in the large cities of the United States. These lectures to be upon medical, surgical, and a few special topics.

The lectures will express not only the personal views of the lecturers upon the subjects treated of, but also the latest pathological and therapeutical opinions connected with these topics, and will therefore be trustworthy guides to practice. It is expected that these lectures, presenting in compact and economical shape the most advanced thought, combined with practical instruction, will be found equally valuable by advanced medical students and by practitioners.

The series will be begun by the publication of one lecture each month, but if sufficient encouragement be received, it is proposed to make the issue semi-monthly.

The lectures will be carefully and handsomely printed in pamphlet form; each number to contain from twenty to thirty octavo pages, which will be so proportioned as to leave ample margin for binding.

For the first year no subscriptions will be received, but the lectures will be sold separately at from 30 to 50 cents each.

The series will be under the editorial control of Dr. E. C. Seguin, who desires to state that he has already received the most encouraging support for the enterprise, and has arranged for early contributions from

Prof. AUSTIN FLINT, Sr., Prof. LEWIS A. SAYRE, Prof. ALFRED L. LOOMIS, Prof. A. JACOBI, Prof. T. G. THOMAS, Prof. W. H. THOMSON, Prof. H. B. SANDS, Prof. W. H. Draper; all of New York.

The first number of the series will be ready about February 1st, and will consist of a lecture by Prof. LEWIS A. SAYRE, on Disease of the Hip-Joint.

In order to direct the attention of the profession, and through them of the public at large, to the claims of a class of population deserving in the highest degree the charity of all, we republish with much pleasure, "an eloquent appeal on behalf of these unfortunates," "Feeble-Minded Children," more especially as the case which constitutes the illustration of the appeal, is personally known to the Junior Editor of the JOURNAL, who was consulted professionally in regard to him, and who advised that he be placed in the Institution, where his improvement has been so decided as to elicit this appeal from the father, a worthy citizen of this city.

We deem the very worthy character of the object, and our personal knowledge of the parties, a sufficient apology for departing from our rule, in republishing matter from secular journals—the "appeal" having first appeared as a communication to the "Editor of the Post and Mail," of this city.

### FEEBLE-MINDED CHILDREN.

### AN ELOQUENT APPEAL ON BEHALF OF THE STATE INSTITUTION FOR THESE UNFORTUNATES.

To the Editor of the Chicago Post and Mail.

SIR—In the Governor's late message he recommends an appropriation for the benefit of the Institution for Feeble-Minded Children, at Jacksonville. There is a bill now under consideration in the House, making such an appropriation. I have a son seven years of age in this institution. For years I have expended large sums of money upon physicians and teachers for him, besides an unlimited amount of care. We have, however, never been able to teach him the alphabet, to play with other children, or to sit properly at the table. Now, after three months at this institution, he does all these things gracefully and well. With this experience I cannot find words to sufficiently commend the institution. I have no doubt, under its care, he will become a useful man. No one can doubt the importance of this work. It cannot be done by parents or private teachers.

They have not the experience or skill such as can be found in an institution set apart for the purpose.

Now, notwithstanding its importance, this institution occupies small wooden tenements liable to fire, and poorly arranged. The buildings have accommodations for only eighty pupils, while there are one hundred in attendance. During its existence of only eight years there have been over six hundred applications for admission.

According to the report of the Illinois Medical Society, a building large enough to equal the demands of the State should have accommodations for at least three hundred pupils. With the success it has already accomplished, in its cramped condition, what could it not do with proper buildings and resources? Why should the State not give the managers an opportunity to extend these blessings to others, and to make feeble-minded children able men and women, rather than consign them to the almshouses for life?

It is my earnest desire that the Legislature will make such an appropriation, and I have no doubt it would return tenfold blessings for the expenditure.

SAMUEL GOLDMAN.

Chicago, Jan. 30, 1875.

### *Institution for the Feeble-Minded.*

MEMORIAL OF THE ILLINOIS STATE MEDICAL ASSOCIATION TO THE TWENTY-NINTH GENERAL ASSEMBLY OF THE STATE OF ILLINOIS.

To the Honorable Senate and House of Representatives of the State of Illinois :

The undersigned would respectfully represent to your honorable body, that at the annual session of the Illinois State Medical Association, an organization extending over the entire State, holden at Chicago, in May, 1874, the following resolution of Dr. J. H. Hollister, of Chicago, now president of the Association, was unanimously adopted:

*Resolved*, That a committee of three, consisting of Drs. W. P. Peirce, of Lemont, Cook county; J. L. White, of Bloomington, McLean county, and E. P. Cook, of Mendota, LaSalle county, shall prepare and present a memorial from the Illinois State Medical Association to the next General Assembly of Illinois, earnestly recommending appropriations to erect buildings for the custody, care and instruction of the idiots and feeble-minded children of the State.

This committee, as in duty bound, have proceeded to make investigation into the condition and wants of the idiotic and feeble-minded among our growing population; to take into consideration their number and present condition; and also, what steps are being taken by the State to place them on a level with those other children of misfortune, which it is the just pride and boast of our State to bring within the arms of its all-embracing charity.

Your petitioners take the liberty to say, that in all enlightened States and countries throughout the civilized world, there is no class to which a more lively sympathy is now being extended than the one we have in view. That it is, indeed, the crowning triumph of the country in which we live, that this class, until lately abandoned as beyond hope of being raised in the scale of humanity, left as the prey of brutish instincts, and to remain as a reproach to the species whose form they wear, can now be humanized, taught and educated, and, as it were, brought back to common tastes, enjoyments and spheres of ordinary usefulness. The astonishing success attending the efforts to raise and instruct this hitherto neglected class, has resulted in the permanent establishment of special institutions for their benefit, that are now universally recognized as at least equal in importance with any others which States feel it a duty and a satisfaction to provide.

Institutions of this class abound in all the leading countries of Europe, and their success forms one of the brightest pages of the progress of humanity and social science.

In our own country, quick to follow the lead, and as we believe, bound to excel the Old World in all that relates to human progress, Massachusetts has already three institutions; New York two, and Connecticut, Pennsylvania, Ohio, Kentucky and Georgia one each, to which is to be added the embryo attempt in our own State; hence the subject is no longer an experiment, but stands approved and sanctioned by the best thought of the age in which we live.

As to the magnitude of this subject, we respectfully refer you to unquestioned statistics.

From returns received by the Secretary of the Board of Public Charities for the State of Illinois, and published in its first biennial report, there were reported to said board, by the physicians of the State, the names of 1,738 idiotic persons as actually then resident in the State. This is much less than the actual number, for the difficulties in the way of gaining accurate information in relation to the defective classes of society are well known.

Without question the idiotic at least equal if not exceed the insane in point of numbers, and it is safe to say that there are now in the State of Illinois 3,000 subjects, who, in the strict medical application of the term, would be classed as idiotic.

The number of those actually fitted for instruction is indicated in the records of the school now in operation in Jacksonville in this State. By these it appears that there are now six hundred and eighty-one recorded applicants for admission to that institution.

When it is reflected that these have the same natural claims for instruction belonging to all the children in the State, that they are excluded from ordinary schools, and must remain uninstructed except as the State specially provides for them, their serious claims for legislative regard become at once manifest. Many obstacles exist to their admission into ordinary schools, for their feebler capacities put them at a disadvantage, and they cannot be classified with other scholars of better aptitudes. Even if they are children of persons in good circumstances, having considerable capacity for instruction, motives of justifiable pride prevent them from permitting them to associate with other children, who so naturally make a sport of their misfortune.

Many of them are found in our almshouses, where their condition is most pitiable, and where, from the almost inevitable liabilities of the case, they become subject to neglect and wrong, in some instances outraging every sentiment of humanity.

Hence neglect and brutish ignorance seem the inevitable lot of this most unhappy class, unless the State includes them among the other objects of its care, like the insane, the blind, and the deaf and dumb, whose claim on the public sympathy is certainly no stronger.

Your petitioners can point with a most pleasing satisfaction to the results obtained from the experiment inaugurated in the year 1865. It has already passed beyond the limits of experiment.

From small beginnings, and by an outlay proportionally small, it has taken a position second to no charitable institution of the State, so far as its sphere of usefulness has been able to extend.

Upon leased premises, with cheap and inconvenient buildings, as a temporary resort must necessarily be, and already filled to their utmost capacity, one hundred and four pupils are congregated, and a much larger number are seeking admission.

No one can visit this institution without being touched with feelings of gratitude, in behalf of a common humanity, that a new door is so successfully opened whereby a class hitherto consigned to mental darkness, may find egress to those higher lights of knowledge, that our State provides for all its children.

We speak the sentiments of a profession more intimately acquainted with all the forms of suffering humanity, and as well informed as any in regard to the requirements of public opinion, when we most respectfully yet strongly urge that the needful appropriations be at once made to put the measure we have in view upon a permanent footing, by the provision of safe buildings and other facilities commensurate with the magnitude of the interests involved.

And thus your petitioners, a committee of the Medical Association of the State of Illinois, ever pray.

[Signed] Wm. P. Peirce, M.D., of Lemont, Cook county.
J. L. White, M.D., of Bloomington, McLean county.
E. P. Cook, M.D., of Mendota, LaSalle county.

